# Sex difference in cerebral atherosclerotic stenosis in Chinese asymptomatic subjects

**DOI:** 10.1016/j.heliyon.2023.e18516

**Published:** 2023-07-21

**Authors:** Xiangkun Tao, Renjie Qiao, Can Liu, Lu Guo, Jingcheng Li, Yulai Kang, Youdong Wei

**Affiliations:** a Psychologic Medicine Science, Chongqing Medical University, Chongqing, China; b Clinical Medicine Science, Chongqing Medical University, Chongqing, China; c Department of Radiology, Daping Hospital, Army Medical University, Chongqing, 400042, China; d Department of Neurology, Daping Hospital, Army Medical University, Chongqing, 400042, China; e Department of Neurology, The First Affiliated Hospital of Chongqing Medical University; Chongqing Key Laboratory of Neurobiology, Chongqing, China

**Keywords:** Sex difference, Atherosclerosis, Stenosis, Cerebral artery, Vascular risk factor

## Abstract

**Background and purpose:**

Sex difference in cerebral atherosclerosis has been noted in previous studies, but the precise characteristics remain incompletely elucidated. This study aims to identify the sex difference in patients with asymptomatic cerebrovascular stenosis.

**Materials and methods:**

The image and clinical data of 1305 consecutive patients who had head and neck computed tomography angiography (CTA) were collected. Fifty hundred and seventy-three patients (287 males) with asymptomatic atherosclerotic stenosis in cerebral arteries were finally included. The stenosis number, distribution, severity and their changes with age were analyzed and compared between males and females. Simple linear regression was used to assess the change in lesions with age.

**Results:**

A total of 2097 stenoses were identified in 573 patients, males had more stenoses than females (3 [2, 5] vs 3 [2, 4], *p*=0.015). The number of stenoses in extracranial arteries was much higher in males (*p* = 0.001). Females had higher percentage of stenosis in anterior (89.6% vs 85.9%, *p* = 0.012) and intracranial arteries (63.3% vs 57.1%, *p* = 0.004) than males. Males had higher percentage of moderate-severe stenosis (5.1% vs 3.2%, *p* = 0.026). Age (*OR* = 1.67; 95% CI 1.24–2.25; *p* < 0.001) and hypertension (*OR* = 2.53; 95% CI 1.24–5.15; *p* = 0.01) were associated with moderate-severe stenosis. In patients over 50 years old, the number of stenoses increased by 1.03 per 10 years (*p* < 0.001), with 0.72 more stenoses in males (*p* = 0.003).

**Conclusions:**

Cerebral atherosclerotic stenosis was different between sexes regarding the distribution, severity and the change pattern with age, which underline the sex specific management in patients with cerebral atherosclerosis.

## 1 Introduction

Accumulating evidence has shown that stroke was more prevalent among males than females. Stroke incidence was 33% higher in males, and males got their first stroke on an average 4.3 years earlier than females [1,2]. The prevalence of cerebrovascular atherosclerosis has also been found different between males and females, although the results remain discrepant. An autopsy study showed that from the fourth to the sixth decade, males have higher frequency and severity of atherosclerosis than females, whereas in populations above 65 years of age, the frequency of atherosclerosis was equal in the two sexes [3]. Previous study reported relative females predominance for intracranial atherosclerotic stenosis (ICAS), while males have predilection for extracranial atherosclerotic stenosis (ECAS) [4]. However, another study stated that males were more likely to have ICAS than females [5]. A study in China found that males were more prone to develop middle cerebral artery stenosis than females [6], whereas in the International Atherosclerosis Project, carotid and vertebral arteries were more often affected in males than in females [7].

Sex difference in the prevalence of cerebral atherosclerosis remains incompletely elucidated. The different races, age and enrolled population may partly lead to this discrepancy. In the present study, we sought to investigate sex difference in the burden and distribution of cerebral atherosclerosis, as well as the changes with age in Chinese hospital-based population with asymptomatic cerebrovascular stenosis.

## 2 Patients and methods

### 2.1 Study population

We reviewed database of head and neck computed tomography angiography (CTA) in our hospital from August 2021 to September 2021, and consecutively collected the database of patients. Demographic characteristics and medical histories were obtained from the patients' medical electronic records. Usually, the subjects requested medical evaluation for possible cerebrovascular diseases because of syncope, headache, numbness of limbs, or other symptoms that were believed in general to be associated with stroke, or just because worried about stroke occurrence due to a family stroke history or the presence of traditional stroke risk factors.

Among the 1305 enrolled patients, patients were excluded if they: (1) < 30 years of age; (2) had acute ischemic or hemorrhagic stroke; (3) had history of previous stroke; (4) had no atherosclerotic stenosis within cerebral arteries (5) suspicion of non-atherosclerotic vasculopathy (e.g., Moyamoya disease, vasculitis, or dissection); based on imaging findings and clinical history. The study protocol conforms to the ethical guidelines of the 1975 Declaration of Helsinki and was approved by the Ethics Committee of Daping Hospital (ethical approval number 2022–344). Written informed consent was obtained from each patient included in this study.

### 2.2 Clinical assessments

A binary sex categorization (male/female) was used in this study, and the sex was assigned at birth (sex at birth) and based solely on the visible external anatomy of a newborn. Hypertension was defined as receiving medication for hypertension or blood pressure >140/90 mmHg on repeated measurements. Diabetes was defined as receiving medication for diabetes, fasting blood glucose level ≥126 mg/dL, or 2-h postprandial blood sugar ≥200 mg/dL. Hyperlipidemia was defined as receiving cholesterol-reducing agents or an overnight fasting cholesterol level >200 mg/dL or low-density lipoprotein ≥130 mg/dL. A smoking history was defined as a self-reported current smoker or a person who had quit smoking <6 months previously. History of coronary heart disease was defined as a known history of myocardial infarction, angina pectoris, and congestive heart failure.

### 2.3 Image analysis

CTA scanning was performed with a 64-channel scanner Brilliance 64 (Philips Medical Systems) with a slice thicknesses of 0.625 mm and a resolution of 512 × 512 pixels (pixel size, 0.391 mm). The scan was enhanced by automatic intravenous injection of iodinated contrast material (Iomeron, 350 mg/mL) at a dose of 1–1.2 mL/kg body weight and an injection rate of 4 mL/s. The scan was initiated individually by tracking the loading bolus with automatic triggering at detected values ≥ 120 Hounsfield Units, covering interest region from the aortic arch level to the whole skull. Image processing and postprocessing were conducted at a dedicated workstation (Advantage, General Electric Medical Systems, Chicago, IL, USA). The presence and degree of vascular stenosis were decided independently by two neuroradiologists. When the judgment of the two readers was inconsistent, a decision was entrusted to a third investigator.

The location of atherosclerosis was based on the following classification system. Intracranial arteries included the distal internal carotid artery (ICA) (cavernous and supraclinoid segments), middle cerebral artery (MCA), anterior cerebral artery (ACA), distal vertebral artery (VA) (intradural V4 segment), basilar artery (BA) and posterior cerebral artery (PCA). Extracranial arteries included the proximal ICA, carotid sinus, and proximal VA (ostium, V2–V3 segments) [8].

Extracranial artery stenosis was measured according to the method used in North American Symptomatic Carotid Endarterectomy Trial (NASCET) [9]: stenosis (%) = (1 − N/D) × 100% (N: the diameter of the narrowest lesion; D: the diameter of the distal normal vessel). Intracranial artery stenosis was measured according to the method used in Warfarin-Aspirin Symptomatic Intracranial Disease Study (WASID) [10]: stenosis (%) = [(Dn − Ds)/Dn) × 100% (Ds, the diameter of the artery at the site of the most severe stenosis; Dn, the diameter of the proximal normal vessel). If the proximal segment was diseased, contingency sites including the distal artery (second choice), and the feeding artery (third choice), were chosen to measure Dn. The degree of stenosis was classified as: mild, ≤49%; moderate, 50–69%; severe, ≥70% [11].

### 2.4 Statistical analysis

Kolmogorov-Smirnov tests were performed on all continuous variables. Normal distribution data was described by mean ± standard deviation, and analyzed using independent samples *t*-test for between-group analysis; non-normal distribution data was described by median and interquartile range (IQR), and Mann-Whitney *U* test was used for between-group comparisons. The categorical variables are represented by numbers (%), and comparisons between groups were made using the Pearson χ2 test or Fisher's exact test. To explore the relationship between age and number of stenoses, a 4-node restricted cubic spline (RCS) was used for mapping, and linear regression models were used to assess differences in amount of stenosis between sexes and changes with age. In addition to the initial model, calibrated models corrected for hypertension, diabetes, hyperlipidemia, coronary heart disease and smoking factors were developed. To identify risk factors for moderate to severe stenosis, we performed logistics regression analysis. All tests were two-sided and *p* < 0.05 was considered statistically significant. All the statistical analyses were conducted in the statistical program R (version 4.1.3, R Foundation for Statistical Computing, Vienna, Austria).

## 3 Results

### 3.1 Demographic characteristics

**Fig. 1:**
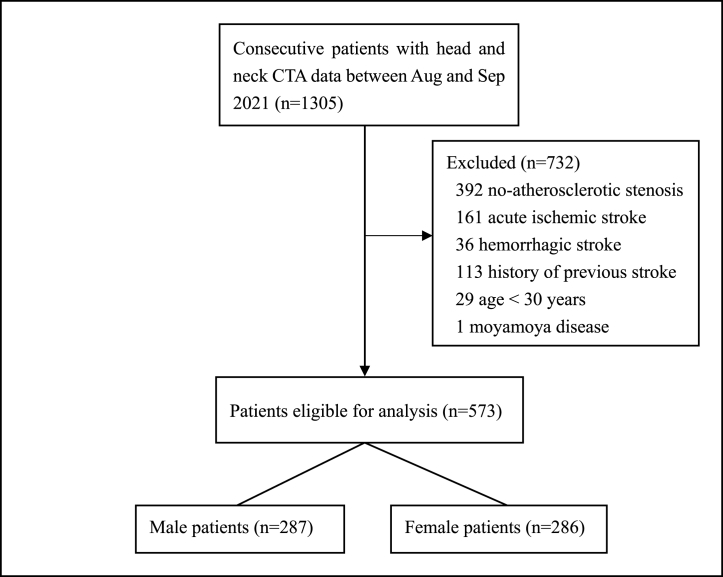
Study Flow Chart; CTA: computed tomography angiography.

Among the 1305 enrolled patients, 732 patients were excluded due to: (1) < 30 years of age; (2) had acute ischemic or hemorrhagic stroke; (3) had history of previous stroke; (4) had no atherosclerotic stenosis within cerebral arteries; (5) Moyamoya disease. The detailed flow chart for patient selection was presented in Fig. 1. A total of 573 patients were included in the final analysis, of which 287 (50.1%) were males (median age, 65 years; range, 36–93 years) and 286 (49.9%) were females (median age, 67 years; range, 33–89 years). In terms of the risk factors, 295 (51.5%) patients had hypertension, 246 (42.9%) had hyperlipidemia, 203 (35.4%) had coronary heart disease, and 112 (19.5%) had history of smoking. Two or more risk factors were found in 302 patients. There was a significant difference in the age composition between males and females (*p* = 0.006). Smoking was more prevalent in males than in females (37.3% vs 1.9%, *p*＜0.001). Comparison of baseline characteristics between males and females was shown in Table 1.

**Table 1:** Baseline characteristics of patients by sex.

Baseline characteristics	Total (573)	Male (287)	Female (286)	*p*-value
Age (years) (IQR)^a^	67 (57,73)	65 (56,74)	67 (59,73)	0.101
Hypertension, n (%)	295 (51.5%)	149 (51.9%)	146 (51.0%)	0.835
Diabetes, n (%)	126 (22.0%)	67 (23.3%)	59 (20.6%)	0.433
Hyperlipidemia, n (%)	246 (42.9%)	121 (42.2%)	125 (43.7%)	0.709
Smoking History, n (%)	112 (19.5%)	107 (37.3%)	5 (1.9%)	<0.001
Coronary heart disease, n (%)	203 (35.4%)	112 (39.0%)	91 (31.8%)	0.071
Number of patients by age				0.006
30–40	7	3	4	
41–50	39	27	12	
51–60	142	80	62	
61–70	185	76	109	
71–80	160	77	83	
>80	40	24	16	

a IQR denotes interquartile range.

### 3.2 Overall distribution of stenoses in males and females

Most patients (81.5% males and 76.9% females) had multiple stenoses in their cerebral arteries. Isolated-anterior circulation stenosis (72.9% [418/573]) was more prevalent than isolated-posterior and combined anterior-posterior stenosis; combined ICAS and ECAS (53.8% [308/573]) was more common than isolated ICAS or ECAS. There was no significant difference in the overall stenosis distribution in anterior and posterior arteries between males and females (*p* = 0.053), nor was there a sex difference (*p* = 0.095) in the distribution in intracranial and extracranial arteries (Supplementary Fig. 1).

### 3.3 Sex difference in the number and distribution of stenoses

The number and distribution of stenoses in two sexes were shown in Table 2. A total of 2097 stenoses were identified, with the distal ICA being the most common lesion site (1097), followed by the sinus portion of the ICA (485) and the proximal ICA (220). For the two sexes, anterior rather than posterior, intracranial rather than extracranial arteries were more often affected. The percentage of stenosis in anterior arteries was even higher in females than in males (89.6% [849 of 948] vs 85.9% [987 of 1149], *p* = 0.012), also the percentage of stenosis in intracranial arteries was significantly higher in females than in males (63.3% [600 of 948] vs 57.1% [656 of 1149], *p* = 0.004). Compared with females, males had higher median number of stenoses (3 [2,5] vs 3 [2,4], *p* = 0.015) in whole cerebral arteries. Higher number of stenoses was found in males in anterior, posterior and extracranial arteries, however, median number of stenoses in intracranial arteries was not statistically different between males and females (2 [1,3] vs 2 [1,3], *p* = 0.299).

**Table 2:** Number of stenoses/moderate-severe stenoses in each portion of cerebral arteries.

Portion of arteries	Any stenoses							Moderate-severe stenoses		
	Total (n = 573)		Male (n = 287)		Female (n = 286)		*p*-value	Total (n = 57)	Male (n = 34)	Female (n = 23)
Whole arteries	2097	3 (2, 5)	1149	3 (2, 5)	948	3 (2, 4)	0.015	89	59	30
Anterior circulation	1836	3 (2, 4)	987	3 (2, 5)	849	3 (2, 4)	0.049	45	28	17
Carotid sinus	485		269		216			10	8	2
Proximal ICA	220		133		87			11	9	2
Distal ICA	1097		566		531			14	7	7
MCA	23		16		7			6	2	4
ACA	11		3		8			4	2	2
Posterior circulation	261	0 (0, 1)	162	0 (0, 1)	99	0 (0, 0)	0.012	44	31	13
VA1	102		68		34			22	16	6
VA2-VA3	34		23		11			3	2	1
VA4	97		57		40			14	10	4
BA	8		5		3			1	1	0
PCA	20		9		11			4	2	2

Grouped according to intracranial and extracranial arteries										

ICAS	1256	2 (1, 3)	656	2 (1, 3)	600	2 (1, 3)	0.299	43	24	19
Anterior-ICAS	1131	2 (1, 3)	585	2 (1, 3)	546	2 (1, 3)	0.476	24	11	13
Posterior-ICAS	125		71		54			19	13	6
ECAS	841	1 (0, 2)	493	1 (0, 2)	348	1 (0, 2)	0.001	46	35	11
Anterior-ECAS	705		402		303			21	17	4
Posterior-ECAS	136		91		45			25	18	7

Abbreviations: ICA: internal carotid artery; MCA: middle cerebral artery; ACA: anterior cerebral artery; VA: vertebral artery; BA: basilar artery; PCA: posterior cerebral artery; ICAS: intracranial atherosclerotic stenosis; ECAS: extracranial atherosclerotic stenosis.

### 3.4 The number and distribution of moderate to severe stenoses in males and females

As shown in Table 2, a total of 89 moderate-severe stenoses were identified. Fifty-nine were found in 34 males and 30 were found in 23 females. The proportion of subjects that have moderate-severe stenoses was not significantly different between males and females (11.8% [34 of 287] vs 8.0% [23 of 286], *p* = 0.128), whereas the percentage of moderate-severe stenosis was higher in males than in females (5.1% [59 of 1149] vs 3.2% [30 of 948], *p* = 0.026). The most common site of moderate-severe stenoses in males was the V1 segment of the VA (16), followed by the V4 segment (10) and the proximal ICA (9), whereas in females the common sites were the distal ICA (7), the V1 segment of the VA (6), the MCA (4) and the V4 segment of the VA (4). The posterior circulation was more likely than anterior circulation to develop moderate-severe stenoses in both males and females; 16.9% (44 of 261) of stenoses in posterior circulation were moderate-severe compared to 2.5% (45 of 1836) in anterior circulation (*p* < 0.001). Age (*OR* = 1.67; 95% CI 1.24–2.25; *p* < 0.001) and hypertension (*OR* = 2.53; 95% CI 1.24–5.15; *p* = 0.01) were associated with moderate-severe stenosis (Supplementary Fig. 2).

### 3.5 Age- and sex-related changes in the number of stenoses

**Fig. 2:**
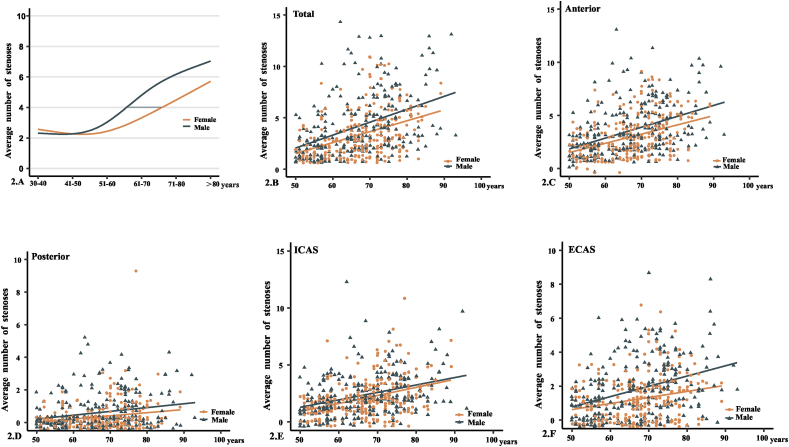
Age-related changes in the number of stenoses in males and females. A. changes in average number of stenoses with age; B–F. in patients aged above 50 years, average number of stenoses grouped by (B) whole cerebral arteries, (C) anterior arteries, (D) posterior arteries, (E) ICAS and (F) ECAS.

As shown in Fig. 2A-F and Table 3, the average number of stenoses increased significantly after the age of 50 years for males and 60 years for females, with females lagging behind males by approximately 10 years. In patients over 50 years old, the number of stenoses increased by 1.18 per 10 years (*p* < 0.001), with 0.84 more stenoses in males than in females (*p* < 0.001); after adjustment for all risk factors, the number of stenoses increased by 1.03 per 10 years (*p* < 0.001), with 0.72 more stenoses in males than in females (*p* = 0.003). The number of stenoses increased in varying degree per 10 years depending on the affected arteries, all of which were statistical difference. Compared with females, males had significantly higher increase of stenosis in anterior, posterior and extracranial arteries, with the exception in intracranial arteries where the increase of stenosis number was not significantly different between sexes (*p* = 0.122). The number of any stenoses and moderate-severe stenoses in each age group by sex were shown in Supplementary Table 1.

**Table 3:** Changes in the number of stenoses after 50 years old.

Groups	Parameter	*β* (95% CI) unadjusted	*p*-values (unadjusted)	*β* (95% CI) adjusted	*p*-values (adjusted)
Total					
	Age, per 10 years	1.180 [0.955, 1.405]	<0.001	1.029 [0.803, 1.256]	<0.001
	Male vs Female	0.843 [0.417, 1.270]	<0.001	0.715 [0.248, 1.181]	0.003
Anterior					
	Age, per 10 years	0.973 [0.799, 1.148]	<0.001	0.867 [0.690, 1.044]	<0.001
	Male vs Female	0.586 [0.254, 0.917]	<0.001	0.490 [0.125, 0.856]	0.008
Posterior					
	Age, per 10 years	0.206 [0.124, 0.289]	<0.001	0.163 [0.079, 0.247]	<0.001
	Male vs Female	0.257 [0.100, 0.414]	0.001	0.224 [0.051, 0.397]	0.011
ICAS					
	Age, per 10 years	0.668 [0.520, 0.816]	<0.001	0.592 [0.442, 0.742]	<0.001
	Male vs Female	0.269 [-0.010, 0.549]	0.059	0.243 [-0.066,0.552]	0.122
ECAS					
	Age, per 10 years	0.512 [0.379, 0.644]	<0.001	0.437 [0.302, 0.572]	<0.001
	Male vs Female	0.574 [0.322, 0.826]	<0.001	0.471 [0.193, 0.750]	0.001

Abbreviations: ICAS: intracranial atherosclerotic stenosis; ECAS: extracranial atherosclerotic stenosis.

## 4 Discussion

Accumulating evidence suggested that males have heavier atherosclerosis burden than females. Atherosclerosis occurs at an earlier age and is more extensive in males. However, sex difference in the severity and distribution of cerebral atherosclerosis has not been fully discussed. The main findings of the present study are as follows: (1) males had more stenoses than females, and developed much more ECAS but not ICAS than females; whereas females were more prone to develop ICAS and anterior stenosis. (2) males had higher percentage of moderate-severe stenosis than females. (3) significant increase in stenosis number occurred 10 years earlier in males, and males also showed more rapid increase in stenosis number than females; intracranial arteries of females were particularly susceptible to age-related atherosclerosis.

Compared with females, males had more stenoses in whole cerebral arteries (*p* = 0.015). Regarding the distribution of cerebral atherosclerosis, notable differences were observed between sexes. Our data showed that females had a higher percentage of anterior stenosis than males (89.6% vs 85.9%, *p* = 0.012). This result was in line with previous study, which noted a much higher ratio of anterior to posterior ICAS in females (3.3) compared with males (1.3) [12]. As to the distribution of stenosis in intracranial and extracranial arteries, males developed much more ECAS but not ICAS than females, while females had significantly higher percentage of ICAS than males (63.3% vs 57.1%, *p* = 0.004) (Table 2), suggesting a particular susceptibility of extracranial arteries for males and intracranial arteries for females. Consistently, a meta-analysis of Asian population showed that female sex was more significantly associated with ICAS rather than ECAS [4]. A study of asymptomatic ICAS in China community population reported that males were associated with a lower prevalence of ICAS, while females were more likely than males to have ICAS in the 40–49 year age group [13]. A multicenter study of acute stroke or transient ischemic attack (TIA) patients noted that females were more likely to have ICAS than males (43.96 vs 34.14%), whereas males had significantly higher percentage of ECAS than females (15.90% vs 10.00%) [14]. Meanwhile, discrepant results have been reported previously. A population-based multicenter study revealed that ICAS prevalence was higher in men (38.5%) than in women (31.7%) [15]. Wityk et al. reported that in patients with acute stroke or TIA, men were more likely to have ICAS than women (29% vs 14%, *p* = 0.03) [5]. Other studies suggested that sex was not associated with ICAS [13,16]. Discrepancies among these studies may be partially attributed to the different disease course and populations. Cerebral atherosclerosis develops earlier in males and steadily progressed with age; whereas females exhibit relatively mild lesions until the sixth decade, with rapidly increasing lesion formation thereafter [16,17]. Besides, studies based on community or hospital populations may lead to inconsistent results, considering that males have higher incidence of stroke and admission rate than females. Additional studies with larger sample sizes across different age groups are needed to better identify this issue.

In this study, 11.8% males and 8.0% females have moderate-severe stenosis, with no difference in the prevalence between two sexes. Compared with females, males had higher percentage of moderate-severe stenosis than females (5.1% vs 3.2%, *p* = 0.026) (Table 2). The risk factors for moderate-severe stenosis have been discussed by previous studies. In stroke or TIA patients with symptomatic ICAS, diabetes, lipid disorder, and metabolic syndrome were significantly more common in patients with severe stenosis than those with moderate stenosis, and lipid disorder was the only independent predictor of severe ICAS [18]. López-Cancio et al. found that in asymptomatic ICAS patients, diabetes and age were independently associated with moderate-severe asymptomatic ICAS [19]. In our study, age and hypertension were associated with moderate-severe stenosis (Supplementary Fig. 2). Moderate-severe stenosis predominantly affected posterior circulation, 16.9% posterior stenosis were moderate-severe stenosis compared to the 2.5% for anterior. Posterior arteries have thinner walls, less elastin, more concentric intima thickening and less vasa vasorum than anterior arteries, which may lead to the progressive deterioration of atherosclerotic lesions, although the precise mechanisms has not yet been clarified [12].

In the present study, the progression of atherosclerosis with aging was different between males and females. The significant increase in stenosis number was observed in males aged over 50 years and females over 60 years, indicating that males experienced stenosis increase in an earlier age than females, which was in consistent with previous reports [17]. Males also showed more rapid increase in stenosis number than females, with 0.72 more stenoses per 10 years (*p* = 0.003). Compared with females, males had significantly higher increase of stenosis in anterior, posterior and extracranial arteries, but not in intracranial arteries, implying a particular susceptibility of intracranial arteries to age-related atherosclerosis in females (Fig. 2, Table 3, Supplementary Table 1). These results were compatible with previous observations. Bae et al. noted that ICAS developed earlier in males, but progressed faster in females with age after the sixth decade [16]. Pu's study found that for the patients with acute stroke or TIA, the distribution of ICAS between men and women had no significant difference in total; however in patients aged >63 years, the incidence of ICAS in women was notably higher than in men (51.51% vs 45.40%; *p* = 0.028) [14]. Compared to extracranial arteries, intracranial arteries have thinner media and adventitia, fewer elastic medial fibers and, especially for women, smaller diameter and less compliant vessel wall, which result in vulnerability of intracranial arteries to age-related hemodynamic changes (e.g., hypertension and arterial tortuosity) and thus atherosclerosis development [20]. However, the precise mechanisms underlying the intracranial arteries to be particularly affected by ageing remains largely unknown.

The potential mechanisms for sex difference in cerebral atherosclerosis remain elusive. One possible explanation is the effects of sex steroids. Cerebrovascular tissues express specific receptors of gonadal steroids, and respond directly to gonadal hormones. Estrogen decreases cerebral vascular tone and increases cerebral blood flow by enhancing endothelial-derived nitric oxide and prostacyclin pathways. In contrast, androgens increase cerebral artery tone. Additionally, estrogen protects vascular from atherosclerosis by increasing mitochondrial efficiency, promoting endothelial cell survival, suppressing vascular smooth muscle migration and stimulating angiogenesis [21]. Clinical study found that short duration of estrogenic lifetime was an independent risk factor for ischemic stroke, with a 51% higher risk for estrogen lifetime exposure of <34 years [22]. These findings suggest that estrogens may protect against ischemic stroke, an effect that seems to cease with menopause. Another explanation is different risk factors between sexes. Blood pressure was found to be higher in males than females of similar ages; moreover, cigarette smoking were more prevalent among males than females [1]. These factors promote the early occurrence of arteriosclerosis in males. For the elderly females, increased risk factors, including diabetes mellitus, hypertension and hyperlipidemia [14], combined with diminished protection of estrogen may lead to rapid deterioration of arteriosclerosis. In particular, elderly females have higher prevalence of diabetes mellitus [23], which has been identified as independent risk factors for ICAS in many studies [24]. Turan's study found that females, especially those with diabetes, were much more likely to develop ICAS in anterior circulation [18]. This may explain the particular susceptibility of intracranial arteries to age-related atherosclerosis in females. The difference in vascular structure between the sexes should also be considered. Females have relatively smaller intracranial arteries, less compliant vessel wall, and higher velocity at the same degree of stenosis, which leads to higher susceptibility to various risk factors especially in their late life [4].

Some limitations need to be considered: (1) We retrospectively recruited subjects with asymptomatic atherosclerotic stenosis in hospital-based population, which may lead to selection bias. Our conclusions may not be applicable to general population. Longitudinal studies enrolled larger community-based population should be conducted to further verify these findings. (2) CTA is a lumenographic imaging methods, some lesions without luminal stenosis may be undetected, which may lead to underestimation of the actual atherosclerosis burden. (3) Some risk factors such as socioeconomic, dietary, exercise and family history were not included in analysis. (4) Other underlying etiologies of stenotic vasculopathy are not likely to be the cause of stenosis in our participants because of their image features and clinical comorbidities. However, it was difficult to exclude other etiologies with certainty using CTA.

In summary, our study demonstrated important sex difference in cerebral artery atherosclerosis. Although further longitudinal studies with larger community-based population are needed to corroborate these findings and explore the potential mechanisms, our study provide detailed data regarding the frequency, severity and changes with age of cerebral atherosclerosis, which may have clinical implications in encouraging sex specific management in patients with cerebral atherosclerosis.

## Financial support

This study was supported by 10.13039/501100001809National Natural Science Foundation of China (NSFC 81771287) and Chongqing Health and Family Planning Commission under grant numbers (2017MSXM023 and 2017ZBXM046).

## Author contribution statement

Xiangkun Tao: Conceived and designed the experiments; Performed the experiments; Analyzed and interpreted the data; Wrote the paper.

Renjie Qiao, Can Liu: Performed the experiments; Analyzed and interpreted the data.

Luo Guo, Jingcheng Li: Analyzed and interpreted the data, Contributed reagents, materials, analysis tools or data.

Yulai Kang, Youdong Wei: Conceived and designed the experiments; Performed the experiments; Wrote the paper.

## Data availability statement

Data will be made available on request.

## Declaration of competing interest

The authors declare that they have no known competing financial interests or personal relationships that could have appeared to influence the work reported in this paper.

## Appendix A Supplementary data

Supplementary Figure 1 Overall distribution of stenosis in males and females. A. grouped by single and multiple stenosis; B. grouped by isolated anterior, isolated posterior and combined anterior and posterior; C. grouped by isolated ICAS, isolated ECAS and combined ICAS and ECAS.. Supplementary Figure 1

Supplementary Figure 2 Risk factors for moderate-severe stenosis. Supplementary Figure 2
