# Supplementary figures and images for: Sex difference in cerebral atherosclerotic stenosis in Chinese asymptomatic subjects
Source: Heliyon. 2023 Jul 21;9(8):e18516. doi: 10.1016/j.heliyon.2023.e18516 (PMC10407042; doi:10.1016/j.heliyon.2023.e18516)

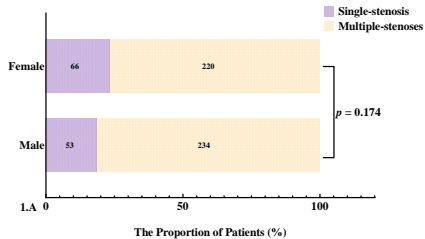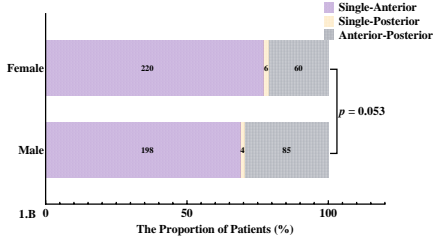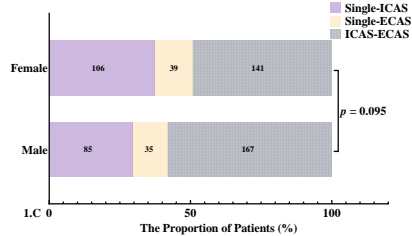

Supplement: Supplementary Figure 1 — Overall distribution of stenosis in males and females. A. grouped by single and multiple stenosis; B. grouped by isolated anterior, isolated posterior and combined anterior and posterior; C. grouped by isolated ICAS, isolated ECAS and combined ICAS and ECAS.. [file mmc1.pdf]

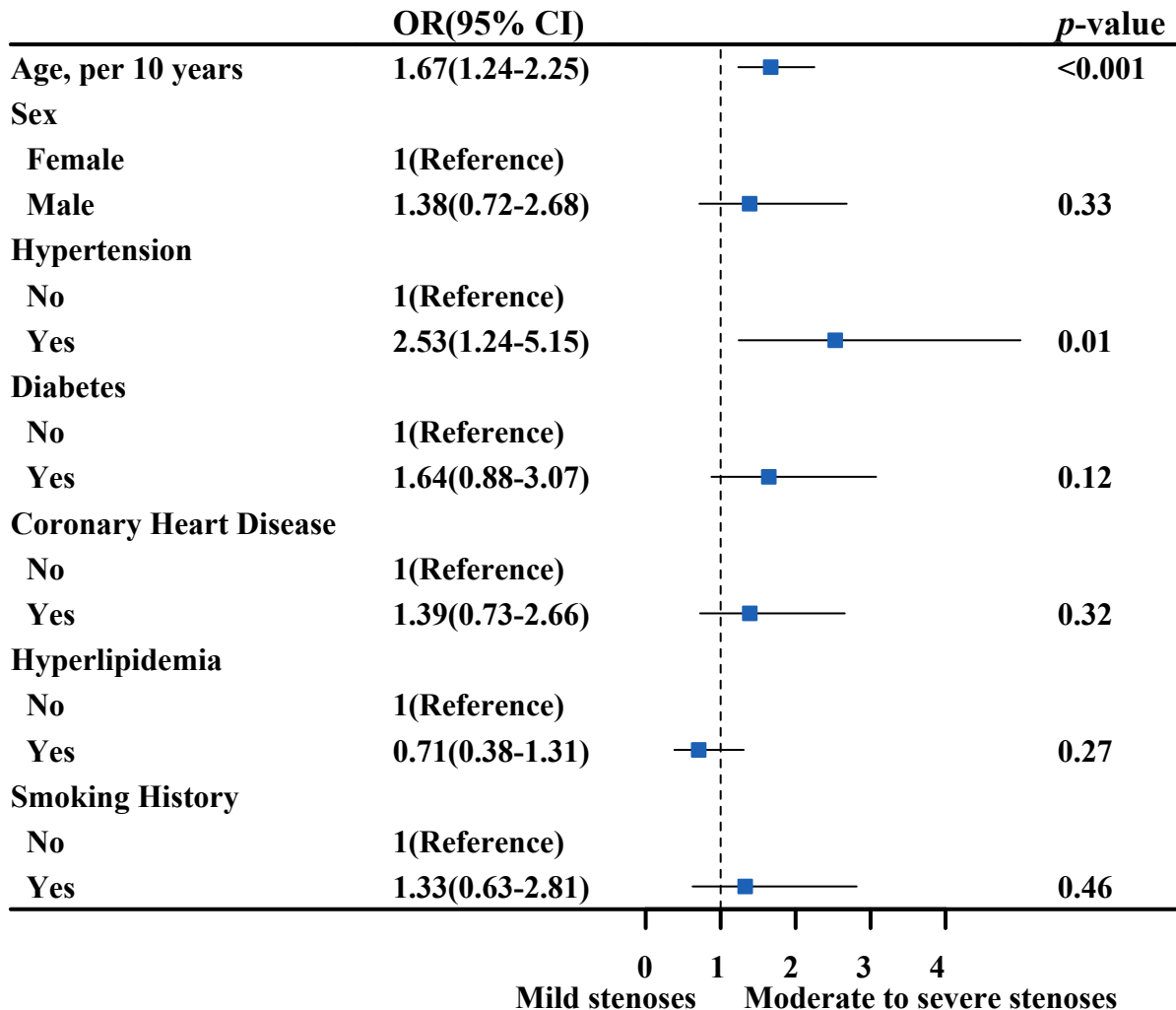

Supplement: Supplementary Figure 2 — Risk factors for moderate-severe stenosis. [file mmc2.pdf]
