# Supplementary material for: Sex difference in cerebral atherosclerotic stenosis in Chinese asymptomatic subjects
Source: Heliyon. 2023 Jul 21;9(8):e18516. doi: 10.1016/j.heliyon.2023.e18516 (PMC10407042; doi:10.1016/j.heliyon.2023.e18516)
Supplement: Multimedia component 3 [file mmc3.docx]

| **Supplementary Table 1** | | | | | | | | | | | |
| --- | --- | --- | --- | --- | --- | --- | --- | --- | --- | --- | --- |
| Number of stenoses/moderate-severe stenoses in each age group by sex | | | | | | | | | | | |
| Male (n=287) | | | | | | Female (n=286) | | | | | |
| Age (n) | Ant | Post | ICAS | ECAS | Total | Age (n) | Ant | Post | ICAS | ECAS | Total |
| > 80 (24) | 130/3 | 26/5 | 91/4 | 65/4 | 156/8 | > 80  (16) | 72/2 | 8/0 | 51/1 | 29/1 | 80/2 |
| 71-80 (77) | 336/9 | 57/11 | 217/7 | 176/13 | 393/20 | 71-80 (83) | 308/7 | 46/5 | 226/7 | 128/5 | 354/12 |
| 61-70 (76) | 278/16 | 51/10 | 177/10 | 152/16 | 329/26 | 61-70 (109) | 313/5 | 30/6 | 216/7 | 127/4 | 343/11 |
| 51-60 (80) | 183/0 | 22/5 | 133/3 | 72/2 | 205/5 | 51-60 (62) | 124/3 | 13/2 | 86/4 | 51/1 | 137/5 |
| 41-50 (27) | 56/0 | 6/0 | 35/0 | 27/0 | 62/0 | 41-50 (12) | 23/0 | 1/0 | 15/0 | 9/0 | 24/0 |
| 30-40 (3) | 4/0 | 0/0 | 3/0 | 1/0 | 4/0 | 30-40 (4) | 9/0 | 1/0 | 6/0 | 4/0 | 10/0 |
| Total | 987/28 | 162/31 | 656/24 | 493/35 | 1149/59 | Total | 849/17 | 99/13 | 600/19 | 348/11 | 948/30 |
| Abbreviations: Ant: anterior circulation stenosis; Post: posterior circulation stenosis; ICAS: intracranial atherosclerotic stenosis; ECAS: extracranial atherosclerotic stenosis | | | | | | | | | | | |
